# Supplementary material for: The Differential Impact of SRC Expression on the Prognosis of Patients with Head and Neck Squamous Cell Carcinoma
Source: Cancers (Basel). 2019 Oct 25;11(11):1644. doi: 10.3390/cancers11111644 (PMC6896085; doi:10.3390/cancers11111644)
Supplement: Supplementary file 1 [file cancers-11-01644-s001.zip › Supplementary Information cancers-582855.pdf]

SUPPLEMENTARY INFORMATION

Differential impact of SRC expression on the prognosis of patients with head and neck squamous cell carcinoma

Francisco Hermida-Prado[1,2]‡, Rocío Granda-Díaz[1,2]‡, Nagore del-Río-Ibisate[1,2]‡, M. Ángeles Villaronga[1,2], Eva Allonca[1,2], Irati Garmendia[2,3], Luis M. Montuenga[2,3], René Rodríguez[1,2], Aitana Vallina[4], César Alvarez-Marcos[1,2], Juan P. Rodrigo[1,2*], Juana M. García-Pedrero[1,2*].

[1] Department of Otolaryngology, Hospital Universitario Central de Asturias and Instituto de Investigación Sanitaria del Principado de Asturias (ISPA); Instituto Universitario de Oncología del Principado de Asturias, University of Oviedo, 33011 Oviedo, Spain.

[2] Ciber de Cáncer, CIBERONC, 28029 Madrid, Spain.

[3] Program in Solid Tumors, Center for Applied Medical Research (CIMA); Department of Pathology, Anatomy and Physiology, University of Navarra; and Navarra's Health Research Institute (IDISNA), 31008 Pamplona, Spain.

[4] Department of Pathology, Hospital Universitario Central de Asturias and Instituto de Investigación Sanitaria del Principado de Asturias (ISPA); Instituto Universitario de Oncología del Principado de Asturias, University of Oviedo, 33011 Oviedo, Spain.

‡These authors contributed equally to this work.

**** Correspondence:***

Juana María García-Pedrero, PhD E-mail: juanagp.finba@gmail.com

Juan P. Rodrigo, PhD E-mail: jprodrigo@uniovi.es

Hospital Universitario Central de Asturias, Edificio FINBA, Lab ORL, Avda Roma s/n 33011 Oviedo, Spain

SUPPLEMENTARY MATERIALS AND METHODS

Western blotting Analysis

Total protein extracts from freshly frozen tissue samples (tumor and patient-matched normal mucosa) were prepared as previously described [59]. The HNSCC-derived cell lines (UT-SCC2, UT-SCC29, UT-SCC38, UT-SCC40 and UT-SCC42B kindly provided by Prof. R. Grenman from Turku University Finland [60] and FaDu from the ATCC) were lysed in Laemmli sample buffer and sonicated before centrifugation. Protein lysates (40 µg) were resolved by SDS–polyacrylamide gel electrophoresis (SDS–PAGE) and transferred subsequently to nitrocellulose membranes (Amersham Protran, GE Healthcare). The membranes were blocked for 1h with Odyssey blocking buffer and incubated overnight with the following specific primary antibodies at 1:1,000 dilution: phospho-SRC (Y419) (Invitrogen # 44-660G), total SRC (Santa Cruz Biotechnology # sc-8056), and Anti-GAPDH clone 6C5 (Merck Millipore # MAB374; at dilution 1:10,000). The IRDye Infrared Fluorescent secondary antibodies IRDye 800CW and IRDye 680RD were used for detection. Membranes were scanned with the Odyssey Fc Dual-Mode Imaging System (LI-COR Biosciences) using the red (700 nm) and green (800 nm) channels and signal density analysis was performed using Image Studio Lite software (LI-COR, Nebraska). Results were normalized to GAPDH as loading control.

SUPPLEMENTARY FIGURES

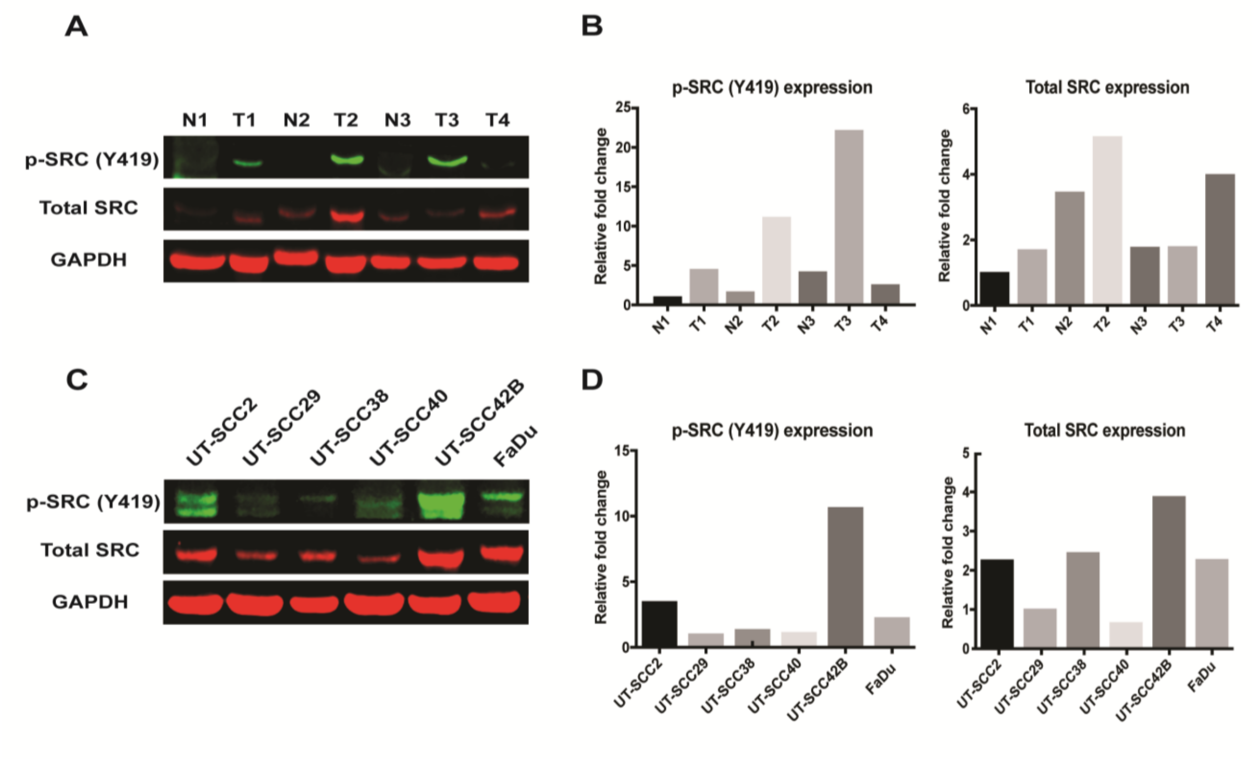

Supplementary Figure S1. (A) Western blot analyses of p-SRC (Tyr419) and total SRC in tumor samples (T) and patient-matched normal mucosa (N) from 4 HNSCC patients. Patients #1-3 are examples of positive expression of active SRC by IHC (scored as 2 and 3, moderate to strong), while patient #4 showed negative expression of active SRC (scored 1, low). GAPDH levels were used as loading control. (B) Quantification of the infrared fluorescent signals from the Western blot analyses shown in A. Data is relative to N1 (C) Western blot analyses of p-SRC (Tyr419) and total SRC in a panel of HNSCC-derived cell lines. (D) Quantification of the infrared fluorescent signals from the Western blot analyses shown in C. Data is relative to UT-SCC29.
